# Optimization of a Quality Improvement Tool for Cancer Diagnosis in Primary Care: Qualitative Study

**DOI:** 10.2196/39277

**Published:** 2022-08-04

**Authors:** Sophie Chima, Javiera Martinez-Gutierrez, Barbara Hunter, Jo-Anne Manski-Nankervis, Jon Emery

**Affiliations:** 1 Centre for Cancer Research Victorian Comprehensive Cancer Centre University of Melbourne Melbourne Australia; 2 Department of General Practice University of Melbourne Melbourne Australia; 3 Department of Family Medicine School of Medicine Pontificia Universidad Católica de Chile Santiago Chile

**Keywords:** cancer, primary health care, diagnosis, quality improvement, clinical decision support tool, general practice, pilot, feasibility, Clinical Performance Feedback Intervention Theory

## Abstract

**Background:**

The most common route to a diagnosis of cancer is through primary care. Delays in diagnosing cancer occur when an opportunity to make a timely diagnosis is missed and is evidenced by patients visiting the general practitioner (GP) on multiple occasions before referral to a specialist. Tools that minimize prolonged diagnostic intervals and reduce missed opportunities to investigate patients for cancer are therefore a priority.

**Objective:**

This study aims to explore the usefulness and feasibility of a novel quality improvement (QI) tool in which algorithms flag abnormal test results that may be indicative of undiagnosed cancer. This study allows for the optimization of the cancer recommendations before testing the efficacy in a randomized controlled trial.

**Methods:**

GPs, practice nurses, practice managers, and consumers were recruited to participate in individual interviews or focus groups. Participants were purposively sampled as part of a pilot and feasibility study, in which primary care practices were receiving recommendations relating to the follow-up of abnormal test results for prostate-specific antigen, thrombocytosis, and iron-deficiency anemia. The Clinical Performance Feedback Intervention Theory (CP-FIT) was applied to the analysis using a thematic approach.

**Results:**

A total of 17 interviews and 3 focus groups (n=18) were completed. Participant themes were mapped to CP-FIT across the constructs of context, recipient, and feedback variables. The key facilitators to use were alignment with workflow, recognized need, the perceived importance of the clinical topic, and the GPs’ perception that the recommendations were within their control. Barriers to use included competing priorities, usability and complexity of the recommendations, and knowledge of the clinical topic. There was consistency between consumer and practitioner perspectives, reporting language concerns associated with the word *cancer*, the need for more patient-facing resources, and time constraints of the consultation to address patients’ worries.

**Conclusions:**

There was a recognized need for the QI tool to support the diagnosis of cancer in primary care, but barriers were identified that hindered the usability and actionability of the recommendations in practice. In response, the tool has been refined and is currently being evaluated as part of a randomized controlled trial. Successful and effective implementation of this QI tool could support the detection of patients at risk of undiagnosed cancer in primary care and assist in preventing unnecessary delays.

## Introduction

The diagnosis of cancer in primary care is complex, owing to the nonspecific nature of many presenting symptoms [1-3]. In particular, symptoms of cancer are often consistent with more common diagnoses [4-6]. This complexity can lead to delays in diagnosis and multiple visits to the general practitioner (GP) before cancer is considered [5], and significantly prolong the primary care interval (from a patient’s first presentation to the GP up to specialist referral) [7,8].

While the factors that influence delays in diagnosis are multifaceted, a timely response to abnormal test results that may herald an underlying cancer can improve patient outcomes and reduce time to diagnosis [9-12]. In primary care, delays can be due to missed opportunities to consider a cancer diagnosis and arrange further investigations [13-15]. For example, over one-third of patients with iron-deficiency anemia are not investigated [16,17], and missed opportunities to investigate for gastrointestinal cancers in the presence of so-called *red flag* symptoms leads to delays [18]. While there is no population screening program for prostate cancer in Australia, rates of testing are high (close to 1.5 million prostate-specific antigen [PSA] tests were ordered in 2017) [19,20]. Controversy and confusion about PSA testing and changing guidelines, including altered thresholds for what is abnormal, all contribute to variable rates of follow-up in men with raised PSA levels [21-23]. Missed opportunities are also relevant in areas of new evidence [24]. Thrombocytosis has recently been identified as an important predictor in primary care for several cancers, including lung and colorectal, but many GPs may be unaware of this new evidence [25-27].

A previous systematic review of computerized decision support systems (CDSSs) to assist with the identification of patients at risk of an undiagnosed cancer found that they have the potential to minimize prolonged diagnostic intervals and reduce missed opportunities to diagnose cancer [28]. Quality improvement (QI) platforms involve a combination of interventions, which can include a CDSS with audit and feedback [29]. The evolution in technology allows for the use of the electronic medical record (EMR) to develop quality measures and to facilitate QI-based audit and feedback [30]. Practice population audit tools are complementary to CDSSs, in which algorithms are linked to a clinical knowledge base and produce patient-specific guideline-based recommendations or prompts for consideration at the point of care (PoC) [24,31].

While the development of QI tools is promising, challenges persist around implementation, especially when designed to identify patients who may be at risk of an undiagnosed cancer [28]. QI tools that are designed with continuous involvement and input from the end users are more likely to be effectively embedded in everyday practice [32]. This study explores the usefulness and feasibility of a novel QI tool using algorithms to identify inadequate follow-up of abnormal test results that could be indicative of an undiagnosed cancer and prompt further investigation.

## Methods

### Ethical Considerations

Ethical approval was granted by the University of Melbourne Human Research Ethics Committee and registered with the Medicine and Dentistry Human Ethics Sub-Committee (Ethics ID 1953614). Participation in the interviews and focus groups were voluntary, and informed consent was obtained from all study participants. Participants who completed an interview received gift vouchers (AU $100) as a reimbursement for their time.

### Participants and Study Design

The development of the QI tool Future Health Today (FHT) has been described elsewhere [33]. In summary, FHT consists of two primary components. The first, a PoC prompt, is a CDSS that provides guideline-based recommendations and is visible upon opening the patient’s medical record (Multimedia Appendix 1). The second component is a web-based portal that contains an audit and recall tool, allowing practice staff to review the FHT recommendations at the practice population level and take steps for recall (Multimedia Appendix 2). Although FHT is designed to manage many different conditions, this study focused on the cancer recommendations. FHT uses EMR data to identify patients who may be at risk of an undiagnosed cancer using the results of abnormal tests (iron-deficiency anemia, raised PSA, and raised platelet counts) and patient information (age, sex, and previous cancer diagnoses). If no appropriate follow-up actions for these markers are identified, guideline-specific recommendations will prompt the GP to review relevant patient symptoms and guide further investigation.

FHT was implemented in 12 primary care practices in Melbourne, Australia as part of an optimization study prior to a cluster randomized controlled trial (RCT) [34]. GPs, general practice nurses (GPNs), and practice managers (PMs) were purposively sampled from 9 of the FHT pilot sites (3 sites did not receive the complete set of cancer algorithms due to limitations associated with pathology data in one EMR system). The PSA and platelet recommendations were released in December 2020, and the anemia recommendations were released in March 2021. Participants had been using FHT with recommendations for chronic kidney disease (CKD) for up to 3 months before the cancer algorithms were introduced. Interviews were conducted from February to May 2021; if the user had not seen the cancer recommendations via the PoC prompt during a consultation, the recommendations were shown using a demo of the tool over Zoom (Zoom Video Communications).

### Data Collection

Semistructured interviews were carried out with GPs, GPNs, and PMs to explore their perspectives on the cancer module and recommendations for improving the tool. In addition, three focus groups were conducted, one with GPs and GPNs, and two with consumers. The general practice focus group explored the audit tool and barriers to use. Consumer focus groups explored their perception of the cancer recommendations, barriers to uptake, and current priorities.

### Data Analysis

**Figure 1:**
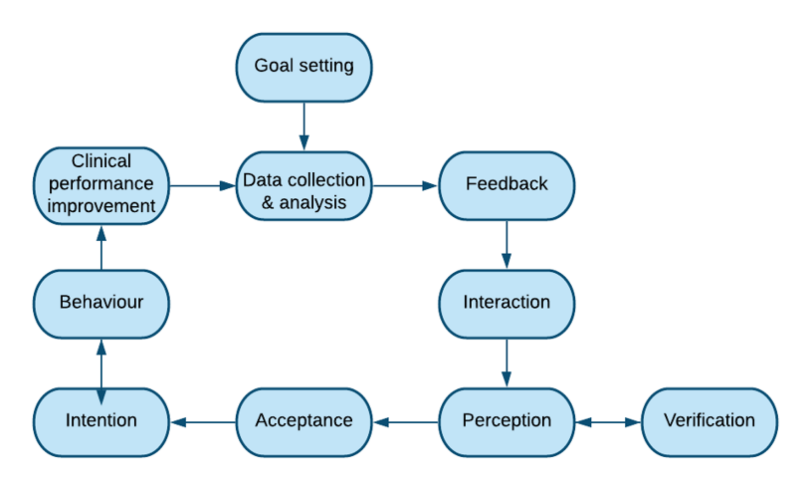
The Clinical Performance Feedback Intervention Theory feedback cycle.

We analyzed all interviews and focus groups using NVivo (version 12; QSR International). The transcripts were independently coded by two reviewers (SC and BH) using an inductive approach to identify themes in the data [35]. Discrepancies in the interpreted data were discussed by the two coders until a consensus was met. For the general practice data, we applied a deductive approach using the Clinical Performance Feedback Intervention Theory (CP-FIT) [36]. A number of frameworks were considered for this analysis. CP-FIT was chosen as it incorporates and builds upon 30 pre-existing theories and, unlike other frameworks, was developed specifically for health care to explain factors that influence feedback success. The theory posits that the feedback cycle (Figure 1) is affected by feedback variables (eg, display and delivery), recipient variables (eg, knowledge of the clinical topic), and context variables (eg, the implementation process) [36]. It describes mechanisms such as compatibility and complexity, which explain how the variables influence the feedback cycle, and resulting clinical performance. In the context of FHT, guideline-based recommendations are communicated to GPs and GPNs. CP-FIT outlines the steps that the user moves through: algorithms are applied to the EMR (data collection and analysis); recommendations are delivered to GPs and GPNs (feedback); and the recommendation is received (interaction), interpreted (perception), and interrogated (verification). If there is acceptance of the recommendation, the user responds to the recommendation (intention and behavior), and ultimately, this leads to changes in patient care (clinical performance improvement) [36].

## Results

We conducted 14 interviews with participants from 6 general practice clinics. As not all participants had experienced the cancer recommendations in practice, follow-up interviews were scheduled with 3 GPs (for a total of 17 interviews). Interviews ranged from 18 to 40 minutes. A total of 8 participants took part in the general practice focus group, of whom 4 had participated in individual interviews. There were 10 participants in the consumer focus groups. Participant characteristics are presented in Table 1.

**Table 1:** Characteristics of the participants and method of data collection

			Interview participants, n (%)		Focus group participants, n (%)
**Role**					
	General practitioner	9 (64)		6 (33)	
	Practice nurse	4 (29)		2 (11)	
	Practice manager	1 (7)		0 (0)	
	Consumer	0 (0)		10 (56)	
**Gender**					
	Female	11 (79)		9 (50)	
	Male	3 (21)		9 (50)	
**Rurality of practice^a^**					
	Metro	7 (50)		6 (75)	
	Regional/rural/remote	7 (50)		2 (35)	

^a^General practice interviews and focus groups only.

### CP-FIT

We mapped practice staff perspectives to CP-FIT. The resulting themes could be mapped across the three constructs: context, feedback, and recipient variables.

#### Context Variables

##### Organization and Team Characteristics: Workflow and Competing Priorities

Most participants reported using the CDSS either before or after the consultation; only 1 GP reported using it exclusively during the consultation. Opening the patient’s file prior to the consultation aligned with the use of FHT, where participants could read, verify, or address the recommendation and, if needed, prepare and print off patient resources or investigation request forms.

In some ways I organise routine care for a consultation before I see the patient, because I won’t remember once they’ve come in and told me all the medicines they need and those kinds of things.

GP2

All practice staff reported competing priorities and an already busy schedule as a barrier to use for all components of the FHT tool.

I haven’t had a lot of time to actually play around with the cohort and implement bringing people in.

PM1

That was coupled with the priorities of the patient as a barrier to being able to address the recommendations during the consultation.

If time allows, I’ll deal with it. It won’t be the first thing because when the patient comes in, they’re coming in because they want to come in.

GP8

While most GPs felt that the recommendations were feasible, some discussed their limited ability to control the consultation and guide the patient to the issues raised by FHT because the patient comes in with their own agenda. This was also discussed in the context of being able to manage the prompts in a single consultation because some recommendations may require long time-intensive conversations to address the patient’s questions and potential worries.

But cancer’s a huge thing - it was much easier to say, by the way, the kidney - this is bad therefore we should do this. Whereas the cancer one is much more nebulous and much more challenging and scary.

GP1

For GPs who had seen the cancer recommendations in practice, half considered delegating some responsibility to either GPNs or medical students. This was driven by their ability to conduct longer consultations and focus on one specific issue raised by FHT.

Their [GPN] ability to engage patients at a level and to quickly put them at ease, I think is quite extraordinary

GP8

##### Patient Population: Clinical Appropriateness and Choice Alignment

Participants were able to determine when the recommendation was not clinically appropriate for their patient. However, the balance of perceived risk (eg, patient distress or risks associated with further investigation) when deciding to investigate for cancer based on the platelet recommendation was raised by two GPs. This may be indicative of a knowledge gap on the association between raised platelets and undiagnosed cancer (see Recipient Variables section).

If you’re going to do a CT of somebody’s chest, you’re clearly exposing them to radiation, what’s the positive likelihood that you’re going to find something if you’re using platelets as a cancer marker?

GP2

Participants frequently framed their reflections on FHT through the lens of their patient population. Some participants saw barriers to actioning the recommendations associated with the patient’s health literacy, language skills, health complexity, and common anxieties associated with the word *cancer*. Others felt that their cohort of patients were well-prepared and responsive to recommendations from the GP.

Our patients are highly health literate. They're very interested in their health. They really are prepared to do something about it. We're very lucky.

GP6

##### Implementation Process: Cost, Training, and Support

While the interviews focused primarily on the cancer module, there were themes around the implementation of the QI tool more broadly, which affected the uptake of the cancer recommendations. For all users, the perceived cost of the CDSS in relation to time was low. Overall, it was reported to be subtle and nondisruptive to the consultation, while the time and resource cost associated with using the audit tool was high and often described as a barrier to use.

I think really useful. It’s all sitting behind in there if you want. It gives you options of having a quick look. It gives you options of re-skilling and it gives you the option of educating the patient as well.

GP3

More than half of the participants reported perceived technical issues, which highlighted areas where more training was needed. For example, to access the audit tool, users needed training on the registration process and errors they may encounter from this process not being completed properly.

I haven’t managed to get my head around exactly how it works [audit tool], so I’m only using the front [CDSS] which pops up on the patient.

GP2

#### Feedback Variables

##### Goal: Importance, Controllability, and Relevance

The themes of importance, relevance, and perceived controllability were present in all participant interviews. The benefits of the intervention were visible to most recipients, and there was a recognized need for the recommendations, which included helpful reminders, reassurance, keeping up with changing guidelines, and ensuring timely action. There was also a recognized need for decision support tools in general.

So, I would be using these tools for re-educating me and ensuring I’m doing best practice because I’m getting older now and things change.

GP3

However, exposure to the recommendations in practice highlighted a shift in priorities. Despite the recognized need for the recommendations, addressing the recommendation became less important and less relevant, as the GPs did not have enough information (see next section) or because of competing priorities.

##### Feedback Display: Usability and Framing

Significant barriers were identified relating to usability. This was due in part to the clarity, length, and language used in the recommendations, which required too much time to process in the time available. The way the recommendations were presented impacted the GP’s ability or desire to engage with the recommendations and indicated a need to modify how we presented the information. Participants reported that the recommendations could be made more concise or were missing essential information (eg, appropriate next steps). Further, it was indicated that the wording of the platelet recommendation was too prescriptive, and therefore, the function of the recommendation did not allow for the GP to exercise their clinical judgement.

In terms of the increased platelets, that’s helpful. What it is though, it’s a bit diffuse in terms of why. Most people are saying, what’s the relationship with thrombocytosis. What cancers does that show, for instance?

GP8

##### Feedback Delivery: Active Delivery, Frequency, and Function

The importance of active delivery was demonstrated in GPs primarily using the CDSS and not using the audit tool (ie, prompts are sent to the users as part of the CDSS, but the audit tool required participants to take steps to obtain the information).

It's not as if it's just not even there and you're never going to see it. When it comes up like that, you know, it might be worthwhile having a look at this and see what's there. It will be a good thing.

PN2

Participants spoke of prompt fatigue in relation to other (non-FHT) prompts or reminders. Participants contrasted these systems with FHT, reporting that they did not find FHT to be an obstruction or an irritant, rather that it had a good balance of appearing when needed, drawing attention but not demanding it, and allowing for action or inaction as the clinician decides.

I do like just it’s a subtle - it’s not in your face that’s coming up all the time as a reminder, because we’ve got so many flashes.

GP3

The GP’s perception of the function of the recommendations was to support them in providing quality care. Most talked about the recommendations as being helpful and beneficial given how much they need to be thinking about, and often framed it is a *suggestion* or *reminder*, indicating that they see it as a supportive rather than a correction or judgement.

I think just to be reminded that a raised platelet count is associated with cancer is a very good thing and I think a lot of GPs wouldn't be aware of it.

GP6

#### Recipient Variables

##### Health Professional Characteristics: The Role and the Knowledge and Skills in the Clinical Topic

There was a difference in uptake of the CDSS and the audit tool by clinical role. Most GPs reported using the CDSS regularly, but only 1 GP reported accessing the audit tool. In comparison, all GPNs had used the audit tool in some capacity where they felt it aligned with their role in the facilitation of patients for recall. However, they did not consider it part of their role to address the cancer recommendations in the PoC. For GPNs, there was a lack of ownership around the content of the recommendations; most stated that the responsibility of doing something about an abnormal test result that a GP had ordered fell solely on the GP. This was an interesting finding, considering the suggestions by some GPs that they would delegate the responsibility of some recommendations to GPNs or medical students (see Context Variables section).

I might do the recalls, I might call people in but in the end, GPs need to be accountable for their work and not me.

PN1

In general, the attitude toward the cancer recommendations was positive both before and after use. Most GPs felt confident acting on the iron-deficiency anemia and PSA recommendations. However, they identified the need for more education to act on the platelet recommendation given that it comes from relatively new evidence. Some GPs felt they did not have adequate knowledge and skills on the clinical topic, which influenced the credibility of these recommendations and the user’s trust in the tool.

I gather from Future Health Today I should be actively looking for occult cancers in this group. But I need – I need a bit more education about it.

GP1

### Consumer Perspectives

The consumer focus groups elicited key themes around language and innate concerns associated with the words *cancer* and *abnormal*. These themes aligned with those identified in the general practice interviews and focus group. Consumers expressed concerns about the short time frame of most consultations coupled with their need to discuss and understand the issues raised by the GP or GPN. There was a consensus around the need for tailored patient resources to aid communication in the consultation and to give patients the ability to digest and review the information in their own time.

### The Feedback Cycle: Which Variables Influence Implementation and How?

**Figure 2:**
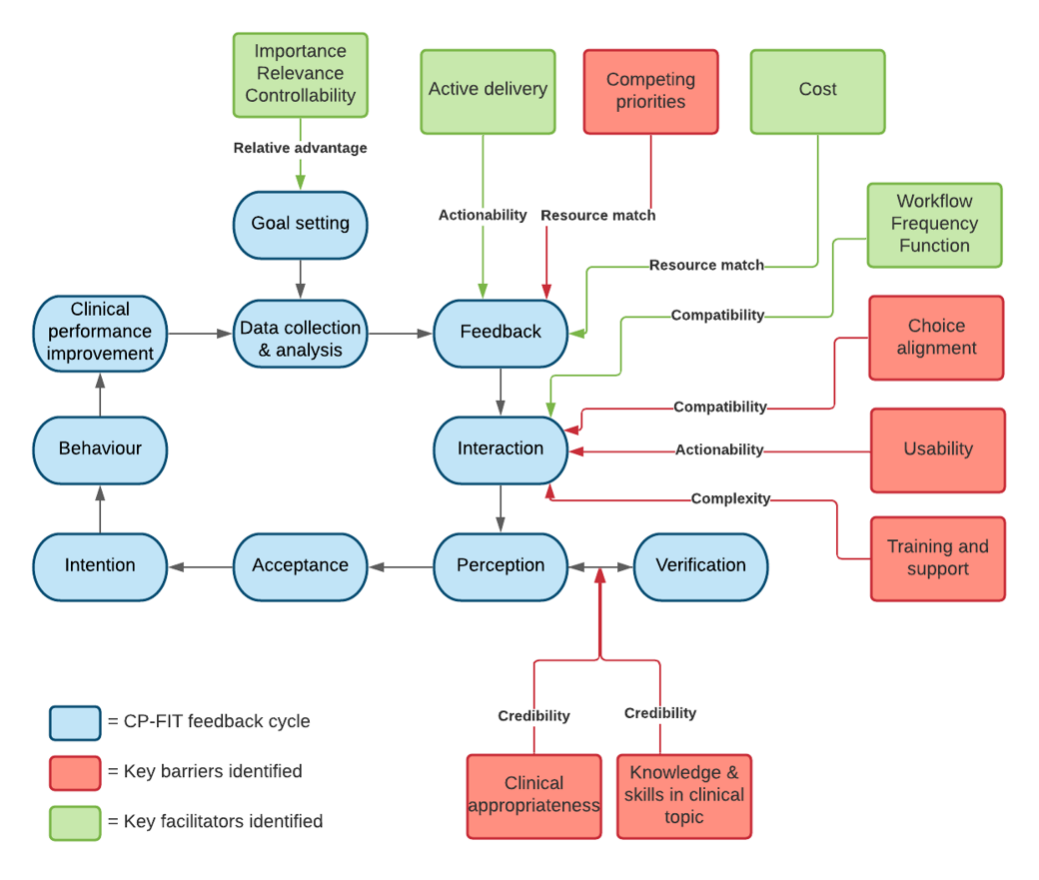
How CP-FIT explains the effectiveness of the Future Health Today intervention. CP-FIT: Clinical Performance Feedback Intervention Theory.

The themes identified as part of this qualitative study can be mapped onto the CP-FIT feedback cycle [36]. Further, the framework analysis allows for the identification of the underlying mechanisms, which provides information as to why and how the platform does, or does not, work as an effective feedback loop. As illustrated in Figure 2, the key components of the feedback cycle where participants were getting *stuck* is the interaction and verification stages. These findings provide an explanation for how FHT for cancer diagnosis in primary care can be improved and guides appropriate action in refining the tool.

## Discussion

### Principal Findings

In this optimization study, participants reported that FHT was easy to use and nondisruptive to the consultation. The cancer recommendations were seen to meet a need that participants recognized and, for most, did not require a change to their consultation style and workflow. However, with repeated exposure to FHT, participants highlighted the complexity that stemmed from the way the cancer recommendations were communicated to users. There was a need to improve the usability and clarity of the recommendations as well as to provide ways for the GP to verify the recommendations. Further, there were barriers when applying the recommendations in practice relating to patient worry, patient communication, and the patient group that the GP saw most often.

We also found that many of the concerns raised by the GP mirrored those raised by consumers. One of the most important although not surprising findings is the importance of language and communication. Both consumers and the GP expressed a need for tailored patient resources to explain why they had recommended further tests and concerns about the time constraints of consultations to address the patient’s worry. The utility of the QI tool for cancer diagnosis relies in the ability to communicate all necessary information accurately, effectively, and concisely in a format that ensures brevity but comprehensiveness at the PoC [37].

By applying CP-FIT as a framework, we were able to illustrate the differences when comparing those who were shown the cancer recommendations at the time of interviewing (ie, a researcher-led simulation) to those who had experience with the recommendations in practice, including those who were reinterviewed after extended exposure. Although the sample size was small, it highlighted in the initial conceptual interviews (when asked to provide immediate feedback on the recommendations) the most prevalent and recurrent themes sat within the *goal* feedback variable at the start of the CP-FIT feedback cycle (ie, importance, relevance, and controllability). Because these variables were met, the response was overwhelmingly positive (there was both acceptance and intention, and the mechanisms indicated actionability and a relative advantage to the way they currently approached these processes). However, once the users had repeated exposure to the recommendations and the users began to move through the feedback cycle (ie, from goal setting to interaction), new barriers were identified (experiential feedback). Although all participants reported competing priorities, this was partially alleviated by ensuring the required time cost was low and the recommendations were actively delivered. However, barriers such as the user’s knowledge of the clinical topic, the usability and clarity of the recommendations, and the need for training and support led to many participants getting stuck at this point in the feedback cycle.

### Limitations

Given the complexities associated with the implementation of QI tools, this study provided an opportunity to evaluate and refine the QI tool for cancer diagnosis with end users. A novel framework was chosen to support the analysis [36]. Participants were recruited from a range of practices, in both rural and metropolitan areas, to ensure a wide range of perspectives. There were, however, significantly more women than men in our sample. While this study targeted GPs and GPNs to explore the clinical appropriateness of the tool and to refine the cancer recommendations, we aim to capture a broader range of perspectives from the primary care workforce in the RCT.

Due to the low frequency with which GPs were exposed to the recommendations, some participants had not seen any cancer recommendations at the time of interviewing and were therefore providing feedback on their expectations of using FHT in practice rather than their actual experience, limiting the generalizability to the usual workflow in the consultation. To address this, we reinterviewed half of the GPs who had not initially seen the recommendations, and this allowed for comparisons of participants’ perception of the recommendations before and after use. The iron-deficiency anemia recommendation was released 3 months after the other recommendations, potentially limiting the amount of feedback on this prompt.

The timing and environment in which this study was conducted is also important. This pilot and feasibility study was conducted during the COVID-19 pandemic, which caused a large disruption to the usual workflow of most primary care professionals. The effects of the pandemic seen in primary care are numerous, with an increase in telehealth appointments, a shift in health perceptions and priorities, and the resulting staff turnover in primary care [38]. We aim to explore how these ongoing changes to usual practice have impacted the use of FHT in the RCT.

### Comparison With Prior Work

While QI tools for cancer diagnosis in primary care are posited to improve the quality of care for patients, reduce practitioner errors, and allow for efficiency in everyday practice, previous studies have reported a range of barriers to implementation and low acceptance in practice. Issues with CDSSs for cancer include tools that are underused [39], too complex [40], incompatible with the workflow [41], incompatible with GP software [42], or do not align with practitioner practice [43]. The results of this study align with the findings of a previous systematic review [28]. The ability to verify the recommendations by understanding the research underpinning the recommendation was not being met as part of FHT [28]. For the diagnosis of cancer, embedding tools in the workflow is often a key barrier [39]; however, the limited disruption caused by the tool and the timing of the prompt meant that FHT aligned with most participants workflow.

The use of a researcher-led simulation in the earlier interviews aligns with previous research that shows that simulations are not able to replicate the stress, workflow, and competing priorities of a usual busy general practice [44]. Nevertheless, they showed a recognized and potentially unmet need around the follow-up of abnormal test results that could be due to an underlying cancer. The later interviews indicated that the perceived usefulness did not translate to optimal usability, and refinements were necessary to address these barriers before testing the efficacy in a large RCT [45]. In particular, changes were made to the language, phrasing, length, and clarity of the recommendations; tailored resources were created to address knowledge gaps; and custom resources were created to address patient communication barriers.

There are implications for further development of this tool. FHT has been developed for use across multiple disease types. The cancer module was implemented in practices after participants had used FHT for CKD. While the tool and its functionality remained constant, the recommendations for CKD showed progression through the feedback cycle (this may be due, in part, to familiarity with CKD guidelines). However, this indicates that the technology has the potential for effective behavior change and improvement in clinical care but highlights that there is no *one size fits all* in the development and messaging of recommendations across disease types. Further work on how to develop, modify, embed, and prioritize these recommendations for use in primary care is needed, especially as the number of conditions within FHT is expanded.

### Conclusions

QI interventions are difficult to implement. This study highlights the benefit of optimization and refinement before testing the efficacy and clinical utility in a large cluster RCT. Successful implementation of this QI tool could be used as a support system to detect patients at risk of an undiagnosed cancer in primary care and assist in reducing diagnostic delays.

## Appendix

Multimedia Appendix 1 An example of the decision support tool, with a recommendation for a patient with raised platelets.

Multimedia Appendix 2 An example of the audit tool, with recommendations for patients with iron-deficiency anemia.

## Abbreviations

CDSS: computerized decision support system
CKD: chronic kidney disease
CP-FIT: Clinical Performance Feedback Intervention Theory
EMR: electronic medical record
FHT: Future Health Today
GP: general practitioner
GPN: general practice nurse
PM: practice manager
PoC: point of care
PSA: prostate-specific antigen
QI: quality improvement
RCT: randomized controlled trial
