# Supplementary figures and images for: Optimization of a Quality Improvement Tool for Cancer Diagnosis in Primary Care: Qualitative Study
Source: JMIR Form Res. 2022 Aug 4;6(8):e39277. doi: 10.2196/39277 (PMC9389376; doi:10.2196/39277)

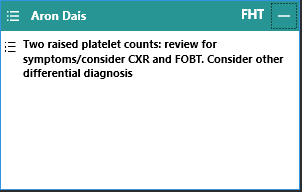

Supplement: Multimedia Appendix 1 [file formative_v6i8e39277_app1.png]

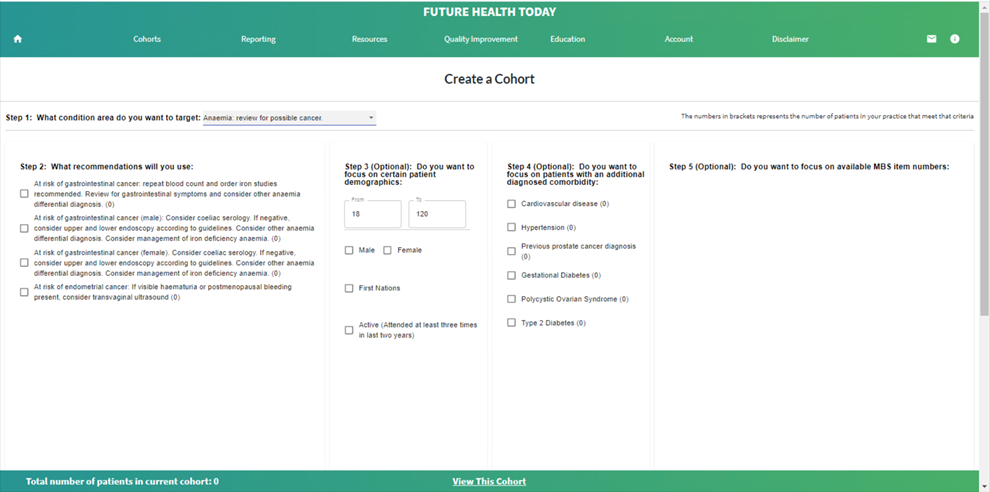

Supplement: Multimedia Appendix 2 [file formative_v6i8e39277_app2.png]
